# Research progress on the relationship between fine motor skills and academic ability in children: a systematic review and meta-analysis

**DOI:** 10.3389/fspor.2024.1386967

**Published:** 2025-01-09

**Authors:** Yucen Li, Xin Wu, Danni Ye, Jinye Zuo, Liu Liu

**Affiliations:** Department of Sports Science, Sichuan University, Chengdu, China

**Keywords:** fine motor skills, academic achievement, preschool children, systematic review, meta-analysis, early childhood education

## Abstract

**Background:**

In recent years, an increasing number of scholars have begun to focus on the relationship between children's motor development and school activities, with the relationship between children's fine motor skills and academic achievement being a particularly researched area. However, due to different research perspectives among scholars, the results in this field have been somewhat controversial. Therefore, this study aims to delve deeper into the relationship between children's fine motor skills and their various academic abilities through systematic review and meta-analysis.

**Method:**

English databases (PubMed, Web of Science, Embase) and Chinese databases (CNKI, Wei Pu) were searched, and a quantitative meta-analysis was conducted using STATA software, along with a systematic descriptive analysis of the included literature.

**Results:**

From the 1,147 documents retrieved, 11 studies were ultimately included. All meta-analysis results are significant, and there is a medium correlation between fine motor skills and reading ability, a larger correlation is observed with mathematical ability. In the subgroup analysis of each fine motor skill component and academic ability, except for the fine motor coordination, which shows only a small correlation with reading ability, the variables in the other subgroups all exhibit a medium degree of correlation. Notably, the correlation between visual-motor integration and mathematical ability is the strongest in subgroup (*r* = 0.47).

**Conclusion:**

The meta-analysis provides evidence supporting a positive and statistically significant correlation between preschool children's fine motor skills and learning outcomes. However, the scope of academic abilities examined in this domain is predominantly confined to mathematics and reading. Moreover, existing research largely focuses on surface-level correlational analyses, necessitating deeper exploration into the underlying mechanisms.

**Systematic Review Registration:**

https://www.crd.york.ac.uk/PROSPERO/, identifier (CRD42023415498).

## 1 Introduction

The early stages of a child's development lay the groundwork for their physical, cognitive, social, and emotional growth, with numerous factors during this period influencing their future achievements (1). Of particular significance is the role played by early motor development in shaping a child's lifelong progress. Motor skills acquired during infancy provide infants and young children with opportunities to engage with the world. Through activities such as crawling, touching, and grasping, children perceive and comprehend their surroundings. Consequently, the development of motor skills offers essential support to cognitive advancement in early childhood (2, 3). Several neuroscience studies have indicated that there is a certain connection between motor and cognitive development; both motor and cognitive activities commonly activate the cerebellum and the dorsolateral prefrontal cortex (4, 5). Motor activities can be broadly classified into two categories: gross motor skills and fine motor skills. Gross motor skills encompass fundamental movements such as walking, running, jumping, and throwing, executed using large muscle groups or body parts (6). Concurrently, cognitive and gross motor skills (fundamental movement skills) share a sensitive developmental phase, with prior research demonstrating a substantial positive correlation between cognitive abilities and gross motor skills (7, 8). Fine motor skills involve precise movements of small muscles, primarily the hands and fingers, to accomplish specific tasks in coordination with psychological processes like perception and attention (2). The development of fine motor skills in children is intricately linked to cognitive enhancement, where the acquisition of fine motor skills lays the foundation for higher-level cognitive activities, while improved cognitive abilities facilitate the refinement of fine motor skills (9–13).

While the relationship between gross motor skills and academic development in young children has been extensively explored, research on fine motor skills remains relatively limited. Nonetheless, recent evidence has indicated that fine motor skills significantly influence a child's later academic capabilities. Pagani et al. extended the predictive model for preschool children originally constructed by Duncan et al. to the second grade of elementary school (1). They found that fine motor skills not only predict children's academic abilities before school age but also effectively predict their academic performance in the second grade (14). This conclusion also indicates that fine motor skills are not only a part of the widely recognized school readiness but are also associated with children's subsequent development. Furthermore, Ricciardi and colleagues conducted a more in-depth study by examining the long-term impact of children's fine motor skills at the age of four on their elementary school academic performance through a large-sample survey. Their research results show that these early fine motor skills still have a significant predictive effect on children's academic achievements in the fifth grade (15). The conclusions of the aforementioned research all indicate that there is a long-term association between fine motor skills and academic ability development, which further underscores the importance of clarifying the relationship between the two.

Furthermore, some scholars have conducted in-depth investigations into specific subcategories of fine motor skills, such as visual perception integration and fine motor coordination. Additionally, varying perspectives among scholars on the link between distinct fine motor skills and academic abilities further accentuate the need for more extensive inquiry in this domain. For example, while Macdonald et al. contend that children's visual perception integration skills significantly correlate with both mathematical and reading abilities (16), the findings of Escolano-Pérez et al. suggest that visual-motor coordination skills exhibit a significant association with reading but not with mathematical abilities (17). Hence, the primary objective of this study was to conduct a rigorous and comprehensive systematic review and meta-analysis of the existing research, utilizing Pearson correlation coefficients as the outcome measure, to assess the interplay between various fine motor skills and diverse academic abilities.

Moreover, this study provides a critical assessment of the current state of research, elucidates encountered challenges, and proposes promising avenues for future investigations. The study also offers novel perspectives and methodological insights for subsequent research in this domain, ultimately providing invaluable guidance for early childhood educational interventions and offering instructive recommendations to enhance preschool children's academic development.

## 2 Materials and methods

### 2.1 Protocol and registration

The study methodology and criteria for inclusion were predefined and registered in PROSPERO (International Prospective Register of Systematic Reviews; Record ID=CRD42023415498). Moreover, the present review adheres strictly to the guidelines outlined in the Preferred Reporting Items for Systematic Reviews and Meta-Analyses (PRISMA) checklist.

### 2.2 Search strategy

A comprehensive and systematic search strategy was employed, utilizing a combination of Medical Subject Headings (MeSH) terms, subject terms, and equivalent keywords. This process yielded a set of 23 key terms, encompassing variations of “Fine motor skill”, “Academic performance”, and relevant terminology, as well as descriptors for “Children” and “Preschool children”. The retrieval method employed in this study involved Boolean retrieval, where subject words were connected using the logical operator “AND”, while free words were linked using the logical operator “OR”. Subsequently, this search strategy was applied across major databases, including PubMed, Web of Science, and EMbase, while similar terms in Chinese were used for searches in the CNKI and WanFang databases. The retrieval logic is shown as follows:

#1 TS = (fine motor skill or fine motor or fine movement or manual dexterity or Manual skill)
#2 TS = (academic performance or performance or test performance or academic or academic test performance or academic test or academic test score or score or test scores or educational test scores or educational test or educational test performance)
#3 TS = (children or child or preschool child or preschool children or primary school student or primary school children)
#4 = (#1 and #2 and #3)

### 2.3 Inclusion criteria

To ensure methodological rigor and relevancy, we employed stringent inclusion criteria. Eligible studies were required to feature participants between 3 and 10 years of age, without any physical developmental disabilities, motor developmental disorders, or intellectual disabilities. Additionally, only cross-sectional or longitudinal studies investigating the correlations between various fine motor skills and academic abilities were considered. The outcomes were restricted to studies reporting Pearson correlation coefficients (*r*) as effect sizes, with complete and robust data. Furthermore, this review encompassed studies published in either English or Chinese, expanding the scope of the relevant literature.

### 2.4 Literature screening

To mitigate bias and uphold scientific integrity, the screening process involved a comprehensive and meticulous procedure. Firstly, the identified duplicate literature was excluded. Subsequently, initial screening based on title, abstract, and citation information was conducted to exclude studies not aligning with the predetermined inclusion criteria. The remaining articles underwent full-text screening to assess their compliance with the specified criteria. Additionally, in cases where the provided information in the literature was incomplete or ambiguous, efforts were made to contact authors for supplementary information or clarification, further enhancing the rigor of the selection process. This systematic screening process yielded a final set of studies meeting the eligibility criteria.

### 2.5 Outcome measures

Upon a thorough examination of the included studies, it was observed that a majority of investigations reported the correlation between fine motor skills and academic per-formance through Pearson correlation coefficients (*r*). A positive value of r indicates a positive correlation between fine motor skills and academic performance. However, some studies presented their findings using Spearman correlation coefficients (*rs*). To maintain consistency in the analysis, Spearman correlation coefficients were uniformly converted to Pearson correlation coefficients using a validated formula, thus facilitating the subsequent meta-analysis (18).

$$rs=\frac{6}{\pi }\sin^{-1}\frac{r}{2}$$

$$r=2\sin\;\left(rs\frac{\pi }{6}\right)$$

### 2.6 Risk of bias assessment

The quality of the included studies was assessed using the 11-item checklist recommended by the Agency for Healthcare Research and Quality (AHRQ). This checklist comprises criteria such as the definition of information sources, inclusion and exclusion criteria, time period and continuity of patient identification, blinding of personnel, quality assurance evaluation, handling of confounding and missing data, patient response rate, and completeness. Each item was scored as “0” if the answer was “unclear” or “no,” and “1” if the answer was “yes.” The quality assessment criteria for this study were as follows: low quality (0–3), moderate quality (4–7), and high quality (8–11). Any disagreements between the two independent assessors were resolved through consultation with a third party.

### 2.7 Statistical methods

The extracted data underwent meta-analysis using Stata 17.0. As the direct combination of Pearson correlation coefficients (*r*) from different studies was not feasible, a conversion formula was employed. Firstly, the extracted *r* values were transformed into Fisher's *Z* scores using the formula. Than, the obtained *Z* values, along with the standard errors (SE) calculated from the Variance of Fisher's *Z* (*Vz*) were put into Stata 17.0 to obtain the summary of Fisher's *Z* value using the inverse variance method. Finally, the summary r value was calculated and assessed for heterogeneity between studies using chi-squared tests. If *P* > 0.1 and *I*^2^ < 50%, this indicates that multiple studies possess homogeneity, and a fixed-effects model can be selected. Conversely, if *P* < 0.1 and *I*^2^ ≥ 50%, a random-effects model can be utilized, Furthermore, when the number of included studies is relatively small (fewer than 4), the Fixed Effects (FE) model is generally considered to be more appropriate (19, 20). The overall correlation between social support and psychological well-being factors was evaluated based on the summary r value. Typically, the absolute value range of the correlation coefficient (*r*) is used to assess the strength of the relationship between variables: 0.5–1.0 represents a large correlation, 0.3–0.5 indicates a medium correlation, 0.1–0.3 suggests a small correlation and 0.0–0.1 represents no correlation (21).

$${\mathrm{Fisher}}^{\prime }s\;Z=0.5\times \ln\frac{1+r}{1-r}$$

$$Vz=\frac{1}{n-3}$$

$$SE=\sqrt{Vz}$$

$$\mathrm{Summary}\;r=\frac{e^{2Z}-1}{e^{2Z}+1}$$

(*Z* is Summary Fisher's *Z* value).

## 3 Results

### 3.1 Literature search

**Figure 1:**
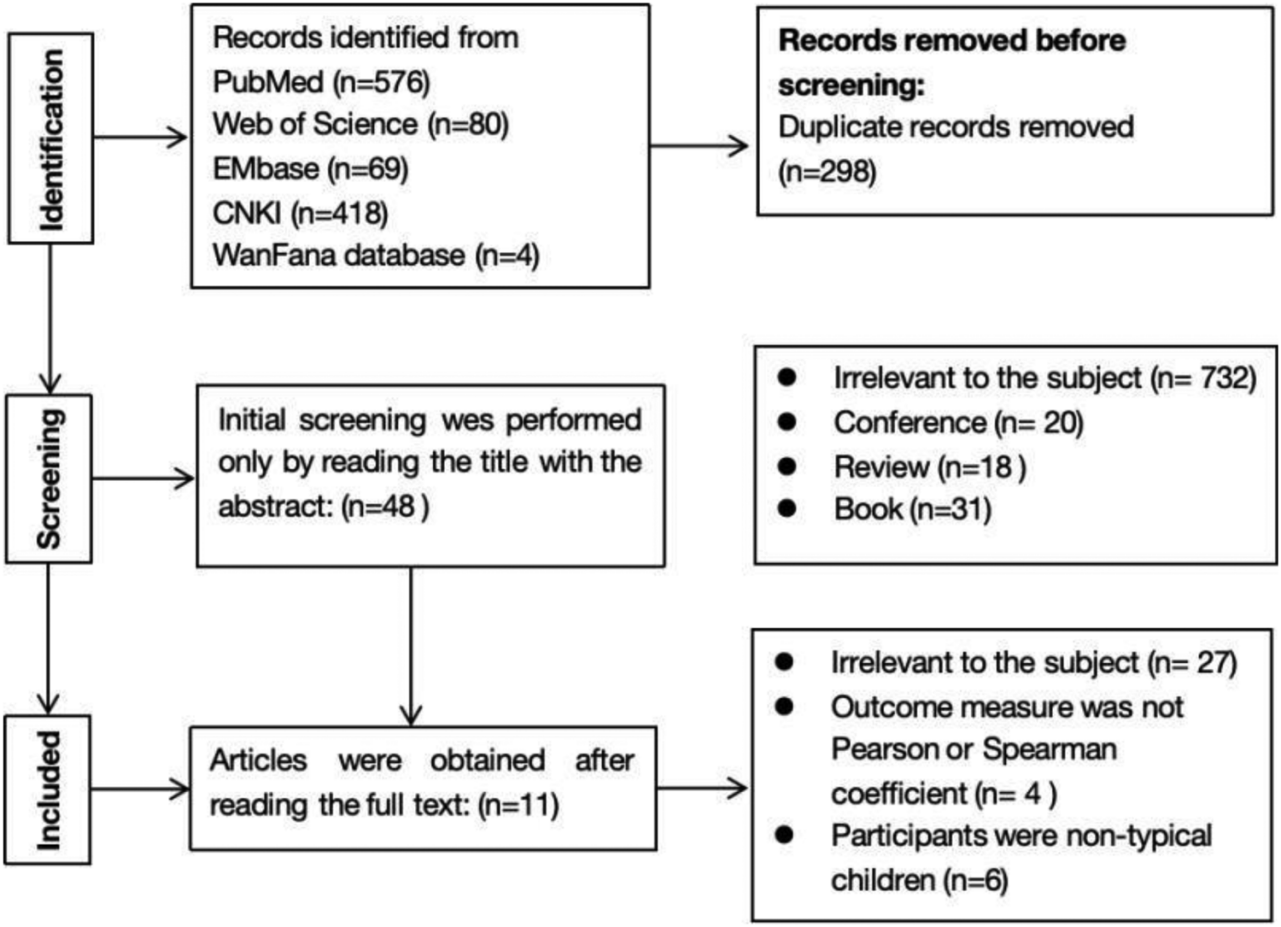
PRISMA flow chart.

The initial database search yielded a total of 1,147 articles, with 576 from PubMed, 80 from Web of Science, 69 from EMbase, 418 from CNKI (China National Knowledge Infrastructure), and 4 from the WanFang database. After removing 298 duplicate articles, a further 69 articles were excluded based on their document types, which included conference papers, reviews, and books. An additional 732 articles were excluded for being irrelevant to the research topic. As a result, 48 articles remained after the initial screening process. Upon obtaining and reading the full texts of these 48 articles, further exclusion was performed based on issues such as lack of data, inability to extract effect sizes, or the inclusion of non-typical children as study subjects. Consequently, 11 articles were included. A flowchart depicting the literature selection process is shown in Figure 1.

### 3.2 Characteristics of included studies

Relevant data and information were extracted from the included articles, focusing on key demographic variables such as sample size, geographical location, family socioeconomic status, and age of the study participants. Additionally, assessment scales for fine motor skills and academic performance were identified, along with the corresponding correlation coefficients between different fine motor skills and various academic abilities.

For the test scale, as illustrated in Table 1, the studies incorporated in this analysis span the years 2013–2020, effectively capturing a recent time frame and demonstrating their contemporaneity. Notably, five of the studies focused on children from the United States, with the deliberate inclusion of participants from diverse racial and ethnic backgrounds. In addition, one study examined the fine motor skills of children in Spain, Australia, the United Kingdom, Germany, and Switzerland. The selected sample pool exhibited a consistent moderate socioeconomic status, contributing to the robust applicability and generalizability of the findings. With regard to the age distribution, all studies, except for a longitudinal exploration that followed participants until the age of 18, encompassed healthy children aged 3–10 years old.

**Table 1:** Characteristics of included literature.

	Year	No. of participants	Country	Economic status	Age	Fine motor skill	Academic ability	Type of study
Carlson (22)	2013	97 (57 M, 40 F)	unclear	unclear	5–18	DTVMI	KTEA-II WJ-III WIAT-II	Longitudinal study
Dinehart and Laura (23)	2013	3,234 (1,530 M, 1,704 F)	Miami, USA	Lower-middle	5.2	LAP-D	SAT-10	Longitudinal study
Escolano-Pérez (17)	2020	38 (12 M, 26 F)	Spain	upper-middle	5–6	BOT-2 MSCA Beery VMI	PAIB-1	Cross-sectional study
Greenburg (24)	2020	34,491	Multi-racial participants	Lower-middle	5–10	Fine motor writing and manipulation scale	FCAT	Longitudinal study
Khng (25)	2021	1,248 (628 M, 620 F)	Multi-racial participants	upper-middle	4.8	IED III	TEMA-3	Cross-sectional study
Macdonald (16)	2020	55 (25M, 30 F)	Australia	unclear	6.77	BOT-2	WIAT-II	Cross-sectional study
Pitchford (9)	2016	62 (29 M, 33 F)	Nottingham, United Kingdom	Lower-middle	6.15	BOT-2	WIAT-II	Cross-sectional study
Roebers (26)	2014	169	Switzerland/Austria	unclear	5.7	MABC-2	HRT-4	Cross-sectional study
Suggate (27)	2019	120	Germany	middle	6	Standardized German version of the Movement-ABC	Dynamic Indicators of Basic Early Literacy Skill	Cross-sectional study
Sulik (28)	2018	343 (176 M, 167 F)	Multi-racial participants	unclear	9.73	ROCF	WISC-IV	Cross-sectional study

Table 1 presents a summary of the findings from the selected articles. Among these, three studies utilized the BOT-2 (Bruininks–Oseretsky Test of Motor Proficiency) scale to evaluate fine motor skills in the study participants. The BOT-2 comprises eight subtests, with the internal consistency reliability for the two subtests relevant to fine motor skills in preschool children reported to be relatively high, ranging from 0.75 to 0.84 in normative samples. The remaining four studies employed different scales, including DTVMI (Developmental Test of Visual Motor Integration) with a reliability coefficient of 0.9; ROCF (Rey–Osterrieth Complex Figure Test) with a reliability coefficient of 0.8, and it's important to note that although the Rey-Osterrieth Complex Figure (ROCF) is not typically used for assessing fine motor skills, the Developmental Scoring System-ROCF (DSS-ROCF) has been shown to effectively evaluate not only visual memory and visual-spatial abilities but also visual-motor integration; LAP-D with reliability coefficients ranging from 0.89 to 0.97 for different subtests, and a fine motor writing and manipulation scale with a reliability coefficient of 0.91 for the writing section and 0.81 for the manipulation section. Regarding the assessment of academic performance, three studies utilized the WIAT-II (Wechsler Individual Achievement Test 2nd Edition) scale, with the age-based item intercorrelation reliability coefficients for mathematics and reading subtests ranging from 0.92 to 0.99 for 5- to 6-year-old children and from 0.79 to 0.98 for 6- to 7-year-old children. The remaining four studies employed different scales, including PAIB-1 (test of basic instrumental aspects: reading, writing, and numeric concepts) with a reliability coefficient of 0.9 for mathematics and 0.89 for language, FCAT (Florida Comprehensive Assessment Test) with a reliability coefficient of 0.88, SAT-10 (Scholastic Aptitude Test) with a reliability coefficient of 0.88, and California state-administered standardized academic achievement tests. In conclusion, the selected assessment scales in the included studies demonstrated satisfactory reliability coefficients, indicating high data reliability for the data extraction process.

### 3.3 Literature quality assessment

In the evaluation of the quality of the included literature using the 11-point checklist recommended by the Agency for Healthcare Research and Quality (AHRQ), the following results were obtained. Except for the studies by Pitchford et al., Nicola et al., Sulik et al., Michael et al., and Escolano-Pérez et al., which were of medium quality, the rest of the included literature exhibited a relatively high quality. The specific evaluation results can be seen in Table 2.

**Table 2:** AHRQ literature quality evaluation results.

	1	2	3	4	5	6	7	8	9	10	11
Escolano-Pérez (17)	Yes	Unclear	Yes	Yes	No	Yes	Yes	Yes	Unclear	Yes	Unclear
Carlson (22)	Yes	Unclear	Yes	Yes	Yes	Unclear	Yes	Yes	Yes	Yes	Unclear
Dinehart (23)	Yes	Unclear	Yes	Yes	Yes	Unclear	Yes	Yes	Yes	Yes	Unclear
Greenburg (24)	Yes	Unclear	Yes	Yes	Yes	Yes	Yes	Yes	Yes	Yes	Unclear
Macdonald (16)	Yes	Unclear	Yes	Yes	Yes	Yes	Yes	Yes	Unclear	Yes	Unclear
Pitchford (9)	Yes	Unclear	Yes	Yes	Yes	Yes	Unclear	No	Yes	Yes	Unclear
Sulik (28)	Yes	Unclear	Yes	Yes	Unclear	Unclear	Yes	Yes	Yes	Yes	Unclear
Suggate (27)	Yes	Unclear	yes	No	Yes	Yes	Yes	Yes	Yes	Yes	Unclear
Khng (25)	Yes	Unclear	yes	No	Yes	Yes	Yes	Yes	Yes	Yes	Unclear
Roebers (26)	Yes	Unclear	yes	No	Yes	Yes	Yes	Yes	Yes	Yes	Unclear

The specific 11 items of the AHRQ are as follows: (1) Is the information source clearly stated (survey, review)? (2) Are exposure group criteria listed or referenced? (3) Is the patient identification period specified? (4) Is the study population consecutive if not population-based? (5) Do raters’ biases affect study subject assessment? (6) Are quality assurance assessments described (e.g., retests)? (7) Are reasons for patient exclusions clarified? (8) Are methods to assess/control confounders described? (9) Are missing data handling methods explained if possible? (10) Is patient response rate and data completeness summarized? (11) If followed up, what's the percentage of incomplete data or follow-up results?.

### 3.4 Results of meta-analysis

We primarily aimed to analyze the correlation between different fine motor skills and mathematics and reading abilities. Although there is some disagreement among different authors regarding the definition of fine motor skills, we also found something in common. Previous definitions can generally be summarized as hand movement skills dominated by small muscle groups, and most researchers primarily classify or define fine motor skills from the following two perspectives: the first is based on the external manifestations of fine movements, such as fine motor manipulation and fine motor writing; the second is the internal function of fine movements, such as visual-motor integration, visual-motor coordination, and fine motor precision. Through the extraction and analysis of the selected literature, we will ultimately conduct a meta-analysis of studies related to visual-motor integration, visual-motor coordination, and fine motor precision. However, the available research on fine-motor manipulation and fine-motor writing is limited, encompassing only one study each, which precluded their amalgamation for a comprehensive meta-analysis. Consequently, to address this limitation, we adopted a descriptive-analytical approach to gain initial insights into the potential relationship between these specific fine motor skills and their influence on mathematics and reading abilities.

#### 3.4.1 Correlation between visual-motor integration and mathematics

**Figure 2:**
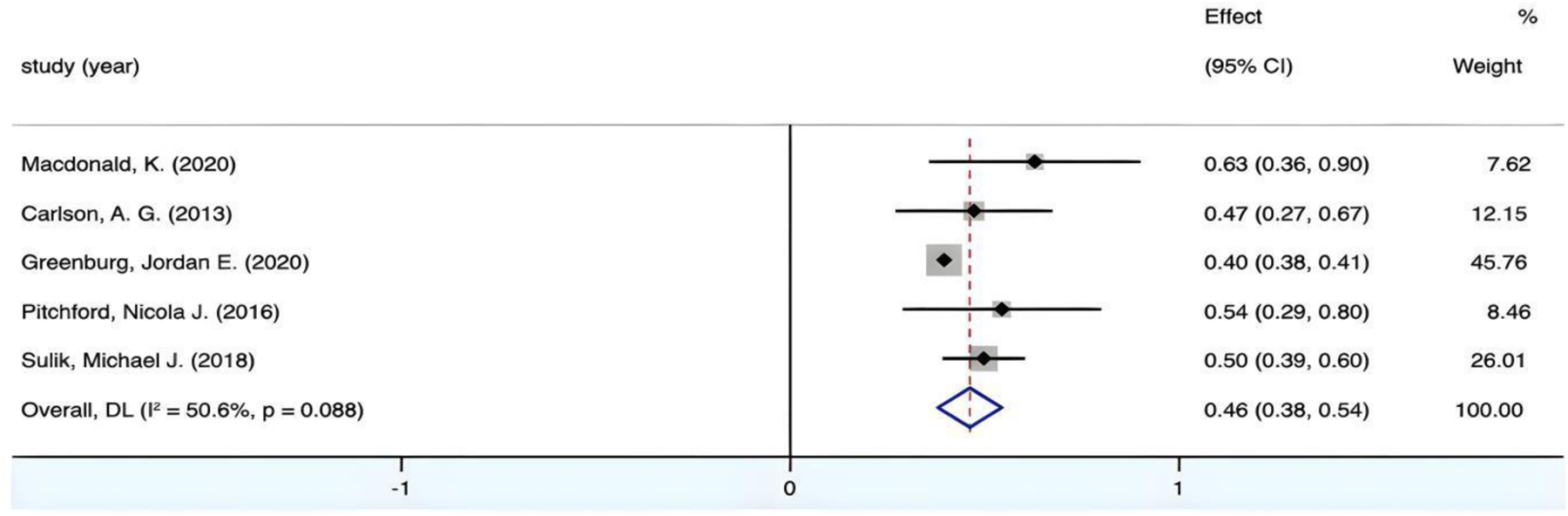
Forest plot of the correlation between VMI and mathematics.

**Figure 3:**
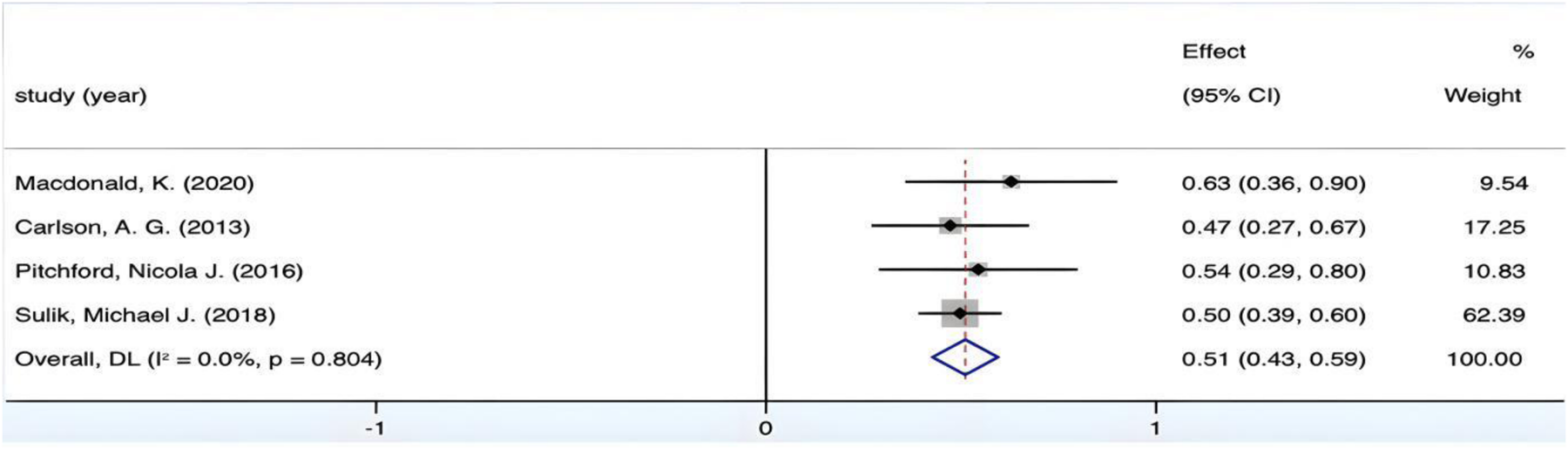
Forest plot of the correlation between VMI and mathematics (after excluding Green-burg's study).

The combined effect size yields a Fisher's *Z* value of 11.018 (*p* < 0.05). Figure 2 displays the *I*^2^ value of 50.6%, indicating considerable heterogeneity among the included studies. To find potential sources of heterogeneity, a sensitivity analysis was conducted, revealing that the study by Greenburg exerted the highest weight in the meta-analysis.Upon analysis, it was found that the study by Greenburg et al. is a longitudinal study, which differs from other studies in terms of research methodology. This further suggests that the study by Greenburg et al. may be a primary inducement of heterogeneity in the meta-analysis. Therefore, we have excluded the study by Greenburg et al. Subsequently, we conducted a heterogeneity test and investigation of the remaining literature using the leave-one-out method to further assess and identify potential sources of heterogeneity. Exclusion of the studies by Macdonald et al., Carlson et al., Pitchford et al., and Sulik et al. was performed one by one, the results still demonstrated statistical significance (Fisher's *Z* values with 95% CI: 0.39–0.53, 0.40–0.54, 0.39–0.53, and 0.38–0.58, respectively). These sequential exclusions confirmed the stability of the overall outcome. Following the removal of Greenburg's study, the combined result (Figure 3) revealed reduced heterogeneity (*I*^2^ < 50%) and a *p*-value greater than 0.01, indicating homogeneity among the studies. Consequently, a fixed-effects model was deemed appropriate for the analysis. The final pooled estimate *r* = 0.47, 95% CI (0.40, 0.53), it indicated a medium positive correlation between visual-motor integration and mathematics development in young children.

#### 3.4.2 Correlation between visual-motor coordination and mathematics

**Figure 4:**
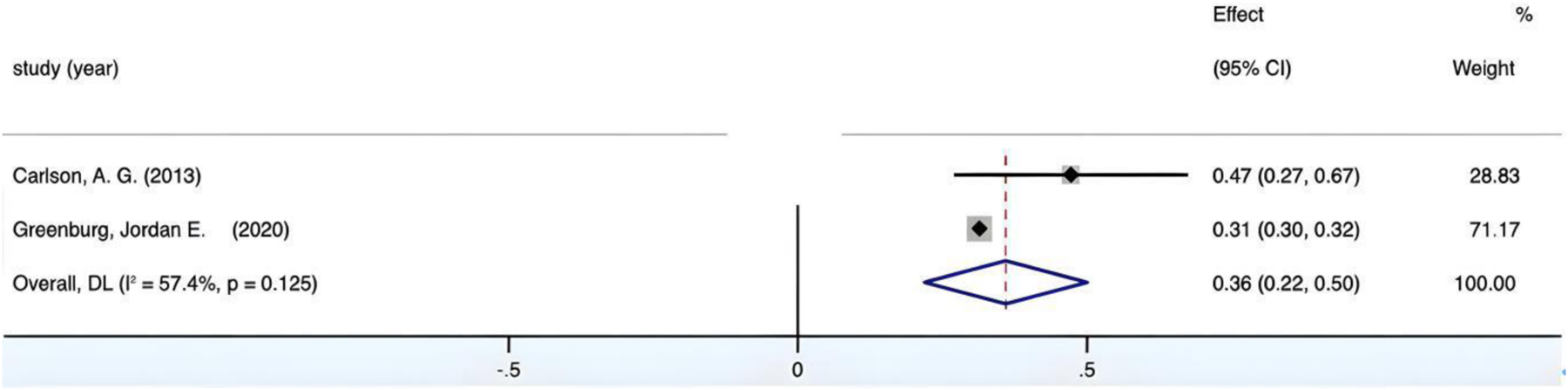
Forest plot of the correlation between VMC and mathematics.

The combined effect size yields a Fisher's *Z* value of 5.014 (*p* < 0.05). However, Figure 4 reveals an *I*^2^ value of 57.4% with a *p*-value surpassing 0.01, suggestive of a degree of heterogeneity across the included studies. Consequently, in light of this observed heterogeneity, a random-effects model was deemed appropriate for conducting the analysis. Upon the final amalgamation of findings, *r* = 0.34, 95% CI (0.22, 0.46), denoting a medium yet positive correlation between fine motor coordination in children and their mathematical ability.

#### 3.4.3 Correlation between fine motor precision and mathematics

**Figure 5:**
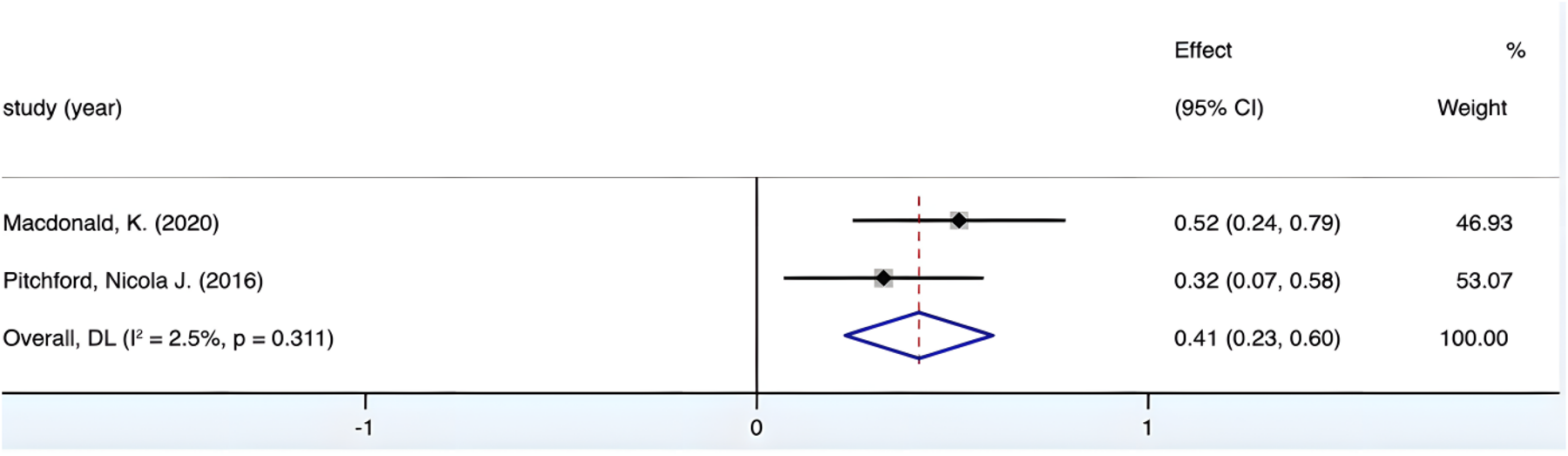
Forest plot of the correlation between fine motor precision and mathematics.

The combined effect size yields a Fisher's *Z* value of 4.319 (*p* < 0.05). Figure 5 demonstrates the *I*^2^ value of only 2.5% with a *p*-value surpassing 0.01, thereby indicating an absence of heterogeneity across the diverse study groups. Given this observed homogeneity, a fixed-effects model was employed to facilitate the analysis. Upon culmination, *r* = 0.39, 95% CI (0.20, 0.54), underscoring a medium correlation between fine motor precision in preschool children and their mathematical proficiency.

#### 3.4.4 Correlation between visual-motor integration and Reading ability

**Figure 6:**
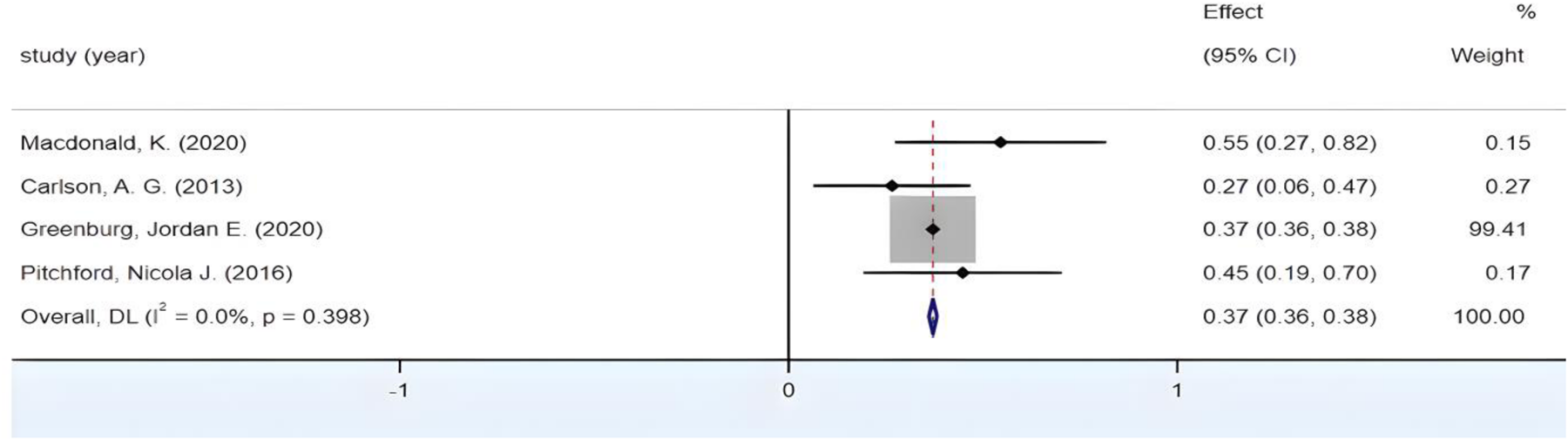
Forest plot of the correlation between VMI and Reading.

**Figure 7:**
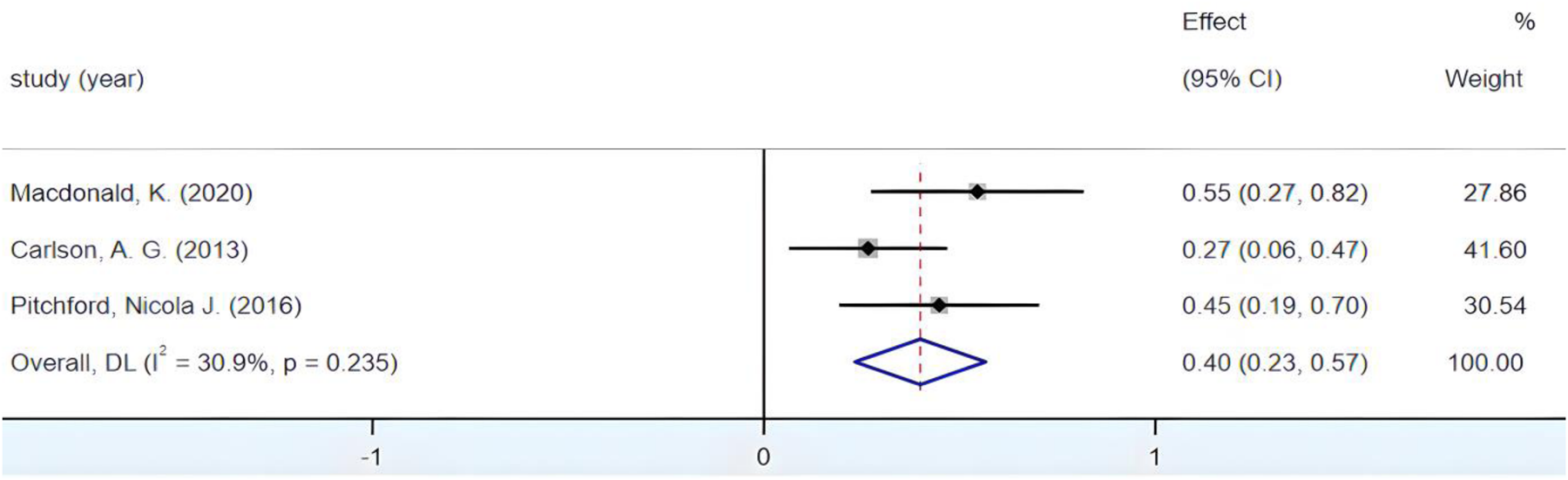
Forest plot of the correlation between VMI and Reading (after excluding Green-burg's study).

The combined effect size yields a Fisher's *Z* value of 69.15, *p* < 0.05, which is statistically significant. Figure 6 shows that *I*^2^ < 50%, *p* > 0.01; In Figure 6, the forest plot, it can be observed that the study of Greenburg et al. constitutes a large portion. Consequently, it is necessary for us to further examine the stability of the analysis results, to see if excluding Greenburg and colleagues’ research would lead to high heterogeneity or insignificant outcomes. Therefore, after the removing the study of Greenburg et al., as shown in Figure 7, *I*^2^ < 50%, *p* > 0.01, the combined Fisher's *Z* still holds statistical significance and still shows no significant heterogeneity between groups. Moreover, we have conducted a leave-one-out method test on the remaining literature to ensure the stability of the results. After excluding the studies by Macdonald et al., Carlson et al., and Pitchford et al. one by one, the 95% CI for Fisher's *Z* are (0.36, 0.38), (0.36, 0.38), and (0.29, 0.45) respectively, all of which are still statistically significant which indicates that the outcome is stable. All of these test show that the presence of the Greenburg study does not affect the heterogeneity between groups, so this study is retained and not excluded. The final combined result is *r* = 0.37, 95% CI (0.36, 0.38), showing that there is a medium positive correlation between visual-motor integration in young children and reading.

#### 3.4.5 Correlation between visual-motor coordination and Reading ability

**Figure 8:**
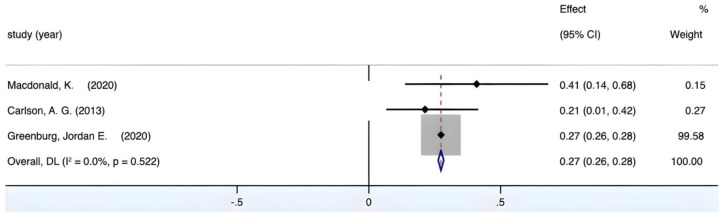
Forest plot of the correlation between VMC and Reading ability.

**Figure 9:**
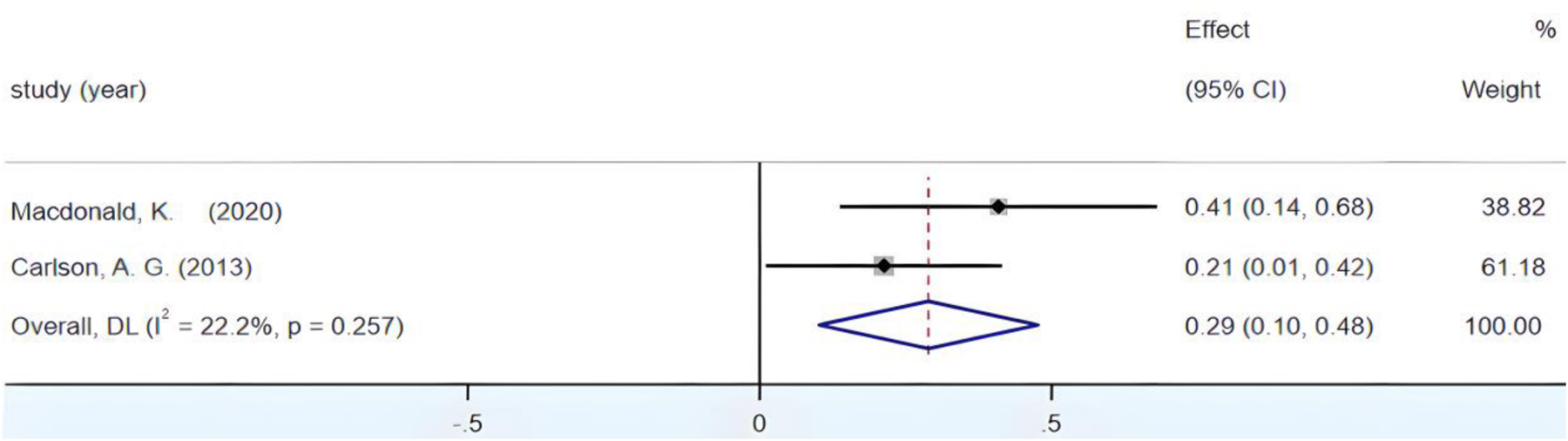
Forest plot of the correlation between VMC and Reading ability (after excluding Greenburg's study).

The combined effect size yields a Fisher's *Z* value of 50.93 (*p* < 0.05). Figure 8 illustrates the *I*^2^ value below the 50% threshold, accompanied by a *p*-value exceeding 0.01. The notable weight ascribed to the Greenburg study created uncertainty regarding the assessment of substantial inter-group heterogeneity. Therefore, a systematic sensitivity analysis of the encompassed literature was essential. During the conducted sensitivity analysis, after the exclusion of the Greenburg study, the Fisher's *Z* at a 95% CI remained within the range (0.10, 0.48), thus persisting in its statistical significance. Sequentially, through the systematic elimination of studies conducted by Macdonald and Carlson, the Fisher's *Z* 95% CIs consistently amounted to (0.263, 0.284), reaffirming the statistical significance. This result underscores the stability of the outcomes. Furthermore, as is evident from Figure 9, following the omission of the Greenburg study, an *I*^2^ value of 22.2% emerged, falling below the 50% threshold, alongside a *p*-value of 0.257, surpassing 0.01. These findings not only corroborate the absence of significant inter-group heterogeneity but also underscore that the inclusion of the Greenburg study does not appear to have a pronounced impact on intergroup heterogeneity. Hence, the decision to retain the Greenburg study in the analysis is soundly supported. Ultimately, the synthesis of the results culminated in an *r* = 0.27, 95% CI (0.26, 0.28), indicative of a small yet positive correlation between children's fine motor coordination and their reading proficiency.

#### 3.4.6 Correlation between fine motor skills and Reading ability

**Figure 10:**
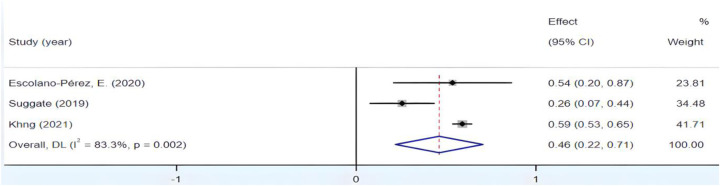
Forest plot of the correlation between fine motor skills and Reading ability.

**Figure 11:**
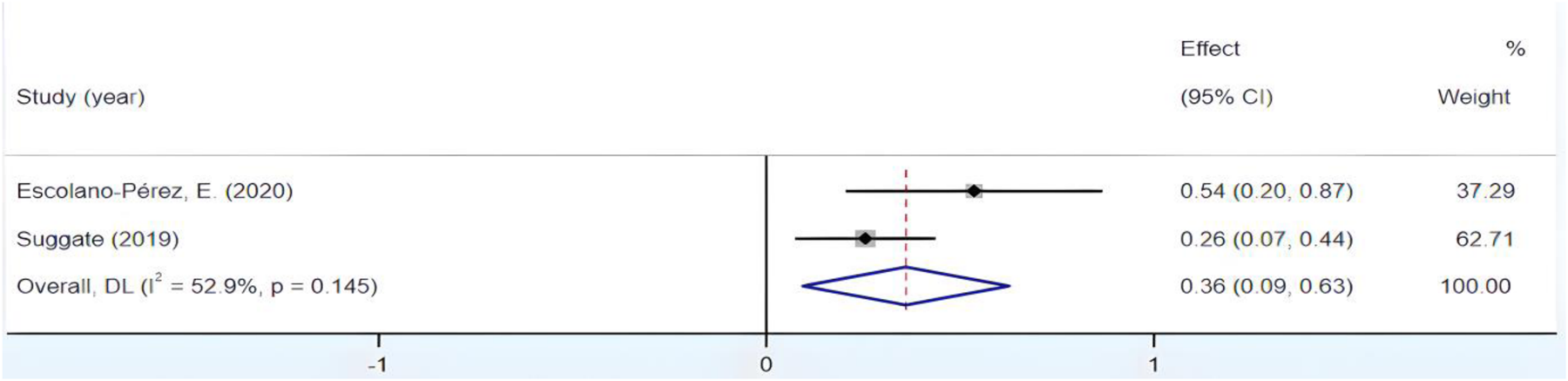
Forest plot of the correlation between fine motor skills and Reading ability (after excluding Khng's study).

Figure 10 reveals significant heterogeneity within the group, with *I*^2^ exceeding 50% and *p* < 0.01. Employing a sensitivity analysis, we initially removed the study by Khng, which held the highest weight, resulting in a notable reduction in heterogeneity, as demonstrated in Figure 11. Subsequently, through successive exclusions of studies by Escolano-Pérez and Suggate, Fisher's *Z* scores at 95% CIs were, respectively, calculated as (0.107, 0.762) and (0.534, 0.643), both of which remained statistically significant. These results indicate the stability of the outcomes. Therefore, the decision to exclude Khng's study was justified. Ultimately, the synthesis of the findings yielded an *r* = 0.35, 95% CI (0.09, 0.56), suggesting a medium yet affirmative correlation between fine motor skills in preschool children and their reading proficiency.

#### 3.4.7 Correlation between fine motor skills and mathematics

**Figure 12:**
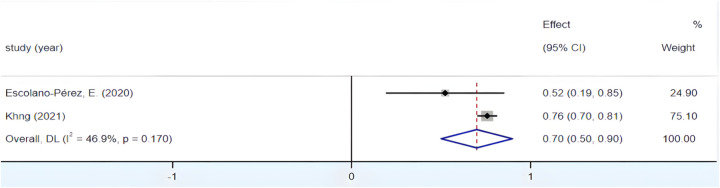
Forest plot of the correlation between fine motor skills and mathematics.

The combined effect size yields a Fisher's *Z* value of 6.879 (*p* < 0.05). Meanwhile, Figure 12 portrays an *I*^2^ value of 46.9%, accompanied by a *p*-value of 0.17, exceeding the 0.01 threshold, implying an absence of inter-group heterogeneity. However, due to the propensity for the adoption of a fixed-effects model to disproportionately amplify the weight of Khng's study, the choice of employing a random-effects model persisted during the meta-analysis. The culmination of this analytical process yielded an integrated *r* = 0.6, 95% CI (0.46, 0.72), underscoring a large and positive correlation between preschoolers' fine motor skills and their mathematical aptitude.

## 4 Discussion

### 4.1 Analysis of reasons for the limited number of included studies

The results of our literature search for this study underscore a prevailing state of relative immaturity, incompleteness, and superficiality within the domain of investigating the relationship between fine motor skills in preschool children and their academic abilities. Despite the rigorous process of literature retrieval and screening, our study succeeded in identifying a mere eleven articles for inclusion. An analysis of the paucity of included studies reveals several contributing factors: Firstly, in the realm of investigating the interplay between motor development and children's cognition, research pertaining to gross motor skills has reached a relatively mature and comprehensive status. Numerous studies have systematically substantiated the intricate connections between gross motor skills, and the multifaceted development of distinct cognitive capacities in children (7, 29–31). Firstly, the emphasis of prior research has largely centered on the fine motor function characteristics of children with physiological and psychological disorders, and cognitive impairments. Consequently, there has been a paucity of investigations delving into the interplay between the developmental patterns of fine motor skills and cognitive growth in typically developing children. Secondly, disagreements among scholars regarding the categorization of fine motor skill structures have emerged, resulting in diverse classifications for research purposes. This divergence in categorization has hindered the depth of exploration within this field and has weakened the continuity between studies.

### 4.2 Disunity in the categorization of the fine motor skill structure

In the extraction of pertinent information from the incorporated literature, an observation emerged that a universally recognized and standardized criterion for classifying fine motor skill structure is conspicuously absent. Different scholars have employed diverse perspectives and methodologies for classification. For instance, Dinehart and Manfra partitioned fine motor skills into fine motor manipulation and fine motor writing, the former pertains to the dexterity of children's hands, involving precise object manipulation capabilities such as grasping, moving, and placing objects, the latter denotes children's capacity to perform writing tasks and graphical representations (23). Conversely, the British scholar Pitchford differentiates fine motor skills into fine motor integration and fine motor precision, the former refers to the ability of children to synchronize hand-eye movements and process visual stimuli, culminating in motor output, encompassing activities like replicating drawings and copying various geometric shapes (9). Fine motor precision entails executing exceedingly delicate manual tasks reliant on minimal visual perception components, exhibited through constrained drawing or paper-folding endeavors. Furthermore, the American scholars Carlson et al. categorize fine motor skills into visual-motor integration and visual-motor coordination. The former predominantly involves fine motor movements of the hand and fingers coupled with visual stimulus processing, prevalent in writing and replication tasks. The latter underscores intricate coordination imbued with visual components, controlling the movement of small fingers, exemplified in diverse sensory-motor tasks such as tracking, finger tapping, imitating hand gestures, and tracing (22). The inherent lack of uniformity in structure categorization has led to a pervasive dearth of continuity, depth, and systematic rigidity in extant research. This incongruence in categorization has, in turn, culminated in our inability to integrate certain outcome indicators during the meta-analysis. As such, the ability to unambiguously define and standardize the classification of fine motor skills is a pivotal issue to be addressed in future research. Clear classification holds promise for facilitating more comprehensive investigations into the interplay between fine motor skills and cognitive development.

### 4.3 The mechanism of fine motor skills' impact on learning abilities awaits exploration

While the meta-analysis has shed light on the correlations between different fine motor skills and various academic abilities, elucidating the precise mechanisms through which fine motor skills influence academic abilities requires in-depth exploration. Neuroscientific evidence suggests that fine motor skills and cognitive abilities might share neuro-logical underpinnings. A study by Li on 5- to 7-year-old children reveals covariation between fine motor skills and cognitive skill levels (32). Their findings suggest an overlap in the temporal and spatial development of early fine motor skills and cognitive development. The seamless development of early fine motor skills could potentially contribute to the maturation of the brain structure and function, consequently promoting cognitive system development. As cognitive performance is closely associated with academic achievement, speculation has arisen among scholars that the influence of fine motor skills on academic abilities may be realized through its impact on cognitive capabilities (33–37). Simultaneously, the study conducted by Cadoret et al. put forth the notion that cognitive ability assumes a mediating role in the intricate relationship between motor skills and early academic performance. To validate this mediation model, a longitudinal study involving 152 children was undertaken. Their ultimate findings of this study substantiated that motor skills exert an indirect influence on academic achievement by virtue of their impact on cognitive abilities (38). Therefore, a thorough investigation into the intricate relationships between fine motor skills, cognitive abilities, and academic performance is warranted. Such exploration will not only provide novel avenues of inquiry but also offer fresh insights for future research. According to the results of this meta-analysis, it can be seen that the correlation between visual motor integration and mathematical ability is the highest. However, there is no research on the interaction between visual integration and mathematical ability, and the underlying mechanisms warrant further investigation in subsequent studies.

In recent years, several scholars have initiated discussions and formulated hypotheses grounded in their research endeavors, shedding light on potential avenues for exploration. For instance, Verdine posits that the connection between visual motor integration and mathematical ability may stem from the effective development of children's spatial imagination abilities facilitated by visual-motor integration (39). Supporting this notion, Sulik's research findings demonstrate a significant predictive relationship between visual-motor integration ability and mathematical development. Sulik further suggests that this predictive capacity may be linked to executive function, with the combined action of visual-motor integration and executive function yielding a more robust predictive influence on mathematical ability (28). These findings are congruent with the hypothesis posited by McClelland and Cameron (40), which advocates for a bidirectional and reciprocal developmental relationship between motor proficiency and cognitive capabilities. Whether this pattern of mutual synergy could be one of the mechanisms by which motor development affects academic abilities is a topic worth exploring in future studies.

It is worth mentioning that not all research conclusions agree that there is a connection between visual-motor integration, executive functions, and mathematical abilities. Early research by Piek and colleagues found that gross motor skills are closely related to cognitive development and are significant predictive factors for cognitive performance such as working memory, which is part of executive functions. However, there was no evidence to suggest that the developmental trajectory of fine motor skills can predict cognitive development (41). More recent research by Duran et al. has proposed that executive functions and visual-motor integration are independent predictive factors for mathematical abilities, and that there is no interaction between visual-motor integration and executive functions in the process of predicting the development of mathematical abilities (34). These diverse viewpoints underscore the complexity of the relationship between visual-motor integration and mathematical ability, and they highlight the need for further empirical research to elucidate the underlying mechanisms and causal pathways involved. Consequently, future investigations in this direction should aim to provide a more comprehensive and nuanced understanding of how visual motor integration contributes to mathematical proficiency.

### 4.4 The impact of visual-motor integration intervention on children's mathematical abilities warrants further in-depth study

The outcomes of the meta-analysis reveal that, in comparison to other subgroups, the correlation between visual-motor integration and early childhood mathematical development is the strongest. Researchers have conducted in-depth explorations into the relationship between these two factors. Pitchford's study corroborates this, highlighting visual-motor integration as a pivotal predictive factor for early mathematical abilities (9). Moreover, Greenburg's subsequent longitudinally inclined study delves deeper, encompassing the tracking of children's academic performance from preschool to elementary school. The results show that children who exhibited robust visual-motor integration capabilities during preschool subsequently outperformed their peers in standardized mathematics tests during third, fourth, and fifth grades (11). This finding offers novel pedagogical implications for educators working with young children. The cultivation of visual-motor integration abilities in preschoolers is potentially efficacious in fostering the early development of mathematical proficiency, thereby narrowing the prevalent academic disparities among children (42). As subsequent research deepens, the relationships between different motor development and different academic abilities are gradually becoming clearer. For example, studies by Verdine and Golinkoff and others have pointed out that mathematics is the academic achievement most related to visual-motor integration (39). Carlson's longitudinal study tracking children from 5 to 18 years old shows that visual-motor integration can effectively explain the variations in children's math scores. Children who scored higher in visual-motor integration tests, even after controlling for gender and socio-economic status, had stronger early math abilities (22). Greenburg's research found that stronger visual-motor integration abilities in preschool education are related to significant improvements in standardized math and reading test scores for children in third, fourth, and fifth grades. This remains true even after adjusting for economic status, and pre-school cognitive, linguistic, and socio-emotional skills (24).

Consequently, how to devise strategies to enhance preschoolers' visual-motor integration skills is the most important question for educators. However, research on methods and approaches for improving visual-motor integration skills is lacking, it is noteworthy that the intervention methods employed in these studies lack uniformity and systematic structure. For instance, in the investigation conducted by Poon et al., their intervention approach involved the utilization of self-developed computer software to enhance children's visuomotor integration ability (43). Similarly, South Korean scholar Minho effectively intervened in the visual-motor integration abilities of individuals with left hemiparesis through Audiovisual Feedback-Based Visual Perceptual Digital Peg-board Training. However, the applicability of this intervention method to typically developing children remains subject to further research and verification (44). Furthermore, as far back as the 1980s, some scholars proposed relevant paradigms, such as Tracking, Copying, and Reproduction (TCR) tasks, for intervening in visual-motor integration abilities. However, due to limitations in research environments and conditions at that time, their studies did not yield conclusive evidence of the effectiveness of TCR training in improving visual-motor integration abilities.

Moreover, there is a notable absence of researchers who have summarized interventions targeting children's visual-motor integration abilities, thereby failing to formulate a comprehensive intervention paradigm systematically. As such, the pursuit of intervention strategies tailored to the unique developmental characteristics and educational environments of the visual-motor integration ability of Chinese preschoolers is a pivotal concern for future research. However, it is not solely visual-motor integration skills that suffer from this deficiency; research into interventions targeting fine motor skills is equally lacking. Knatauskaite once explored intervention methods for fine motor skill development in a study comparing Cardiovascular Exercise (CVE) with Coordinative Exercise (CE). He hypothesized that coordinative training might positively influence attention, subsequently benefiting the development of fine motor skills. However, his research ultimately failed to substantiate a positive impact of CE training on fine motor skill development (45). Therefore, the conformity of intervention methods aimed at enhancing children's fine motor skills and the establishment of a comprehensive intervention framework represent crucial focal points in the realm of research on child motor development and cognitive advancement.

### 4.5 Broader exploration required for the relationship between different academic abilities and fine motor skills

Although the meta-analysis results indicate that visual-motor coordination, fine motor precision and academic abilities exhibit only a moderately low level of correlation, the positive correlation also implies that educational interventions in early childhood should not overlook the development of visual-motor coordination and fine motor precision. A comprehensive approach to fostering fine motor skill development in preschoolers is imperative. Furthermore, given that the current research primarily focuses on preschool-aged children, it is essential to broaden the scope by including children from different age ranges in subsequent investigations. Current studies predominantly assess the academic abilities of preschoolers through mathematical and reading assessments, this is attributed to the cognitive-developmental characteristics of preschool children, where mathematical and reading skills are reliable indicators of cognitive development and academic proficiency (25, 46–48). Additionally, mathematical and reading abilities form the foundational pillars for future interdisciplinary learning and comprehensive academic development. However, in future research focused on older children, apart from mathematical and reading abilities, the relationships between fine motor skills and various academic subjects are also important and should be explored further.

## 5 Conclusions

The outcomes of the current study highlight a positive relationship between the fine motor s kills of preschool children and their academic proficiency, with particular emphasis on the salience of visual-motor integration in relation to mathematical aptitude. This underscores the need for educators focusing on early childhood to address the cultivation of fine motor skills through pedagogical interventions. However, the realm of inquiry in this field is beset with certain gaps that warrant consideration and future investigation:

(1) Lack of Terminology Consensus: A conspicuous void persists concerning the establishment of a uniform and unambiguous lexicon for describing the various facets of fine motor skills.
(2) The experimental methodology requires enhancement to ensure greater scientific rigor: more in-depth empirical researches and experiments are necessary in this field to comprehensively investigate the impact of the development of fine motor skills on children's cognitive abilities and academic performance
(3) Researching Gaps and incomplete methods in intervention frameworks: The lack of comprehensive interventions aimed at enhancing fine motor skills reflects an insufficiency in consolidating and systematizing these interventions into a coherent pedagogical framework.
(4) Age Variability and Academic Breadth: The focus predominantly on preschool subjects raises the need for extending investigations to encompass broader age spectra. Furthermore, the exploration of academic domains beyond mathematics and literacy merits in-depth exploration.
(5) The mechanism of fine motor skills affecting academic ability needs to be further explored: To ascertain the intricate causal mechanisms through which fine motor skills impact academic achievement demands rigorous and probing exploration. In view of the inherent limitations intrinsic to this study, the findings offer insightful perspectives that can catalyze subsequent empirical inquiries. Thus, we anticipate that these results will serve as a compass to guide and stimulate the trajectory of research endeavors within this burgeoning field.

This study's search was restricted to five subsets of databases, potentially introducing the risk of incomplete retrieval. Future research could enhance the search strategy by including a broader array of databases. In addition, the relative scarcity of research in this domain, coupled with the limited number of eligible studies for the me-ta-analysis, necessitated the reliance on a descriptive analysis for the research, which could not be merged. Due to the constrained volume of included literature, the feasibility of conducting a publication bias assessment was compromised. Consequently, the amalgamated findings may be susceptible to biases that cannot be fully evaluated.

## Data Availability

The original contributions presented in the study are included in the article/Supplementary Material, further inquiries can be directed to the corresponding author.
